# Protein–RNA Interaction Links Fragile X Syndrome and Alzheimer Disease

**DOI:** 10.1371/journal.pbio.0050084

**Published:** 2007-02-13

**Authors:** Richard Robinson

No two brain diseases would seem to be more different than Alzheimer disease (AD) and fragile X syndrome (FXS). AD affects the old, while FXS occurs in childhood. AD affects about 4 million Americans of both sexes, while FXS affects fewer than 50,000, primarily boys. And while the cause of most cases of AD is unknown, the gene responsible for virtually every case of FXS has been known for over a decade.

Despite these dissimilarities, the two diseases may be more closely linked than expected. A new study by Cara Westmark and James Malter shows that the protein whose absence causes FXS is a key translation regulator of a protein implicated in AD.

AD, the most common neurodegenerative disease, is characterized by death of neurons throughout the brain. A prominent feature of brain degeneration in most cases is the appearance of amyloid plaques. These sticky clumps are largely made of a protein fragment called beta-amyloid (Aβ). Aβ is cleaved off of a larger protein, amyloid precursor protein (APP), whose precise function is unknown but which is believed to be involved in synapse formation during development. After APP messenger RNA (mRNA) is synthesized, a fraction of it remains quiescent until the neuron is excited by the neurotransmitter glutamate. When that occurs, APP synthesis rapidly spikes upward.

FXS, the most common cause of inherited mental retardation, occurs when a section of the *fmr-1* gene, already containing multiple repeats of the nucleotides cytosine-guanine-guanine, expands to more than 200 repeats. This inhibits transcription of the gene. The gene is on the X chromosome, meaning boys only have one copy. When the mutated and silenced form is the only copy, the result is FXS, which causes—in addition to mental retardation—autistic-like behavior, seizures, and a group of abnormal physical traits. The *fmr-1* gene encodes a protein called FMRP, whose normal role is to bind mRNA and repress its translation. To date, over 500 mRNA targets of FMRP have been identified, many of which may be involved with synaptic function.

FMRP binds with its target mRNAs at sites rich in guanine, especially those whose RNA can fold into a cage-like structure called a guanine quartet (G-quartet). Therefore, the authors began their search for likely FMRP targets by focusing on genes with these sequence characteristics. Their database searches revealed that APP contains such a sequence, which suggested it might be regulated by FMRP. To show that FMRP actually binds to APP mRNA, they isolated synaptoneurosomes, which contain pre- and post-synaptic vesicles, from the mouse brain and treated them with antibodies against FMRP. When the antibodies were isolated, the researchers found they had captured not only FMRP, but also APP mRNA, indicating the two were physically linked together, at least indirectly.

**Figure:**
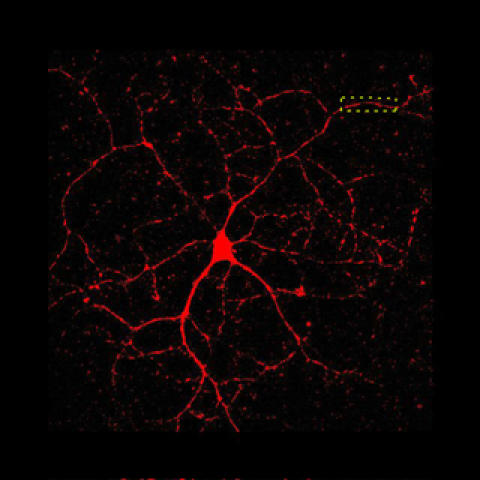
Hot spots in nerve cells are lit up with amyloid precursor protein, the parent molecule that is chopped up to liberate beta-amyloid, which accumulates in Alzheimer disease. (Image: Cara J. Westmark)

They next used neurons in culture to examine the effect of FMRP on the expression of APP. They found that both normal mice and mice missing the *fmr-1* gene (“knockout” mice) expressed APP, but only normal mice increased expression in response to DHPG, which stimulates the glutamate receptor. Thus, it appears that FMRP regulates APP production in some manner. The results were not due to any effect of FMRP on APP mRNA stability, the authors found. Instead, as expected, FMRP repressed APP translation. To show this, the authors treated stimulated synaptoneurosomes with anti-FMRP antibodies. Unlike in resting synaptoneurosomes, in which APP mRNA is isolated along with FMRP, in stimulated synaptoneurosomes, the APP mRNA was not captured, indicating it had dislodged. Presumably, this rapid dissociation of the two is the first step in the stimulus-dependent synthesis of APP.

To determine whether FMRP binds directly to APP mRNA, and not to some other intermediary, the authors digested the FMRP–mRNA complex with RNAse, which digests exposed RNA while passing over the region bound by protein. Their results showed that the two did bind directly with each other, but to their surprise, the FMRP bound a guanine-rich region near, but not identical with, the G-quartet region.

Finally, the authors showed that the brains of *fmr-1* knockout mice, which make no FMRP, contain up to 2.8-fold more Aβ than those of normal mice, in keeping with the loss of translational repression. The significance of this result for FXS patients is unknown. It suggests patients may be at risk for increased amyloid deposits, but very few aged brains from FXS patients have been analyzed, and no deposits have been observed in younger brains. Intriguingly, relatives of FXS patients with only moderately expanded *fmr-1* genes are at risk for developing a late-onset syndrome of dementia and movement disorder (fragile X tremor/ataxia syndrome). Whether this is a direct result of decreased APP repression is unknown as of yet.

Taken together, these results indicate a close relationship between FMRP and APP, with FMRP regulating the normal excitation-dependent expression of APP. Given APP’s probable role in synaptic development, it seems likely that its dysregulation may be a key step in the development of mental retardation in FXS. How, or whether, this relationship also plays a part in AD is much less certain. Nonetheless, given APP’s role in plaque formation, further understanding of how APP is created is likely to lead to new insights into how levels of Aβ are controlled, and from that, perhaps even how those levels might be reduced.

